# Mutual medication in capuchin monkeys – Social anointing improves coverage of topically applied anti-parasite medicines

**DOI:** 10.1038/srep15030

**Published:** 2015-10-12

**Authors:** Mark Bowler, Emily J. E. Messer, Nicolas Claidière, Andrew Whiten

**Affiliations:** 1 School of Psychology & Neuroscience, University of St Andrews, St Andrews, Scotland, United Kingdom; 2 San Diego Zoo Global Institute for Conservation Research, 15600 San Pasqual Valley Road, Escondido, CA 92027-700, USA; 3 Aix Marseille Université, Centre National de la Recherche Scientifique, Laboratoire de Psychologie Cognitive, Unité Mixte de Recherche 7290, Marseille, France; 4 School of Life Sciences Heriot Watt University, Edinburgh, Scotland, United Kingdom

## Abstract

Wild and captive capuchin monkeys will anoint themselves with a range of strong smelling substances including millipedes, ants, limes and onions. Hypotheses for the function of the behaviour range from medicinal to social. However, capuchin monkeys may anoint in contact with other individuals, as well as individually. The function of social anointing has also been explained as either medicinal or to enhance social bonding. By manipulating the abundance of an anointing resource given to two groups of tufted capuchins, we tested predictions derived from the main hypotheses for the functions of anointing and in particular, social anointing. Monkeys engaged in individual and social anointing in similar proportions when resources were rare or common, and monkeys holding resources continued to join anointing groups, indicating that social anointing has functions beyond that of gaining access to resources. The distribution of individual and social anointing actions on the monkeys’ bodies supports a medicinal function for both individual and social anointing, that requires no additional social bonding hypotheses. Individual anointing targets hard-to-see body parts that are harder to groom, whilst social anointing targets hard-to-reach body parts. Social anointing in capuchins is a form of mutual medication that improves coverage of topically applied anti-parasite medicines.

Anointing behaviours, in which animals rub strong smelling substances into their fur, have been recorded in a wide range of animals, including canids^1^, hedgehogs and tenrecs^2^, and several bird species, in which the behaviour is often refereed to as ‘anting’, since ants are most commonly used^3^. In primates, anointing has been recorded in black lemurs (*Eulemur macaco*) with toxic millepedes^4^, black-handed spider monkeys (*Ateles geoffroyi*) with the leaves of aromatic tree species^5^,^6^, orangutans (*Pongo pygmaeus*) with *Commelina* herbs^7^, golden-headed lion tamarins (*Leontopithecus chrysomelas*) with tree exudates^8^ and owl monkeys (*Aotus* spp.) with plants and millipedes^9^,^10^. Most notably, both wild and captive capuchin monkeys (gracile *Cebus* spp and tufted *Sapajus* spp; we follow Silva, 2001^11^; Alfaro *et al.* 2012^12^ in using a separate genus *‘Sapajus’* for the tufted capuchins previously considered *Cebus apella sspp*^13^) anoint with a wide range of materials including plants, aromatic *Piper* spp. leaves, onion (*Alium* spp.), citrus (*Citrus* spp.) fruits, and invertebrates, most commonly ants and millipedes^14^.

Whilst some hypotheses for the function of anointing in non-primates have included social signalling^1^, and sexual selection^15^,^16^, the most common explanations can be described as medicinal^17^. Similarly, anointing in primates is often proposed to function as self-medication against skin parasites^18^ or as a repellent for flying hematophagous insects^19^. These hypotheses are supported by the actions of substances that primates use to anoint in the wild and in captivity. Benzoquinone secretions from millipedes repel insects^19^,^20^,^21^ and ticks^22^, formic acid from ants repels tick nymphs^23^, *Piper* plant leaves are traditionally used by people in Latin America to treat skin conditions^24^, onion (*Allium cepa)* oils kill cattle ticks (*Boophilus annulatus)*^25^ and contain affective antimicrobial agents^26^, and compounds found in citrus fruit peel repel lone star ticks (*Amblyomma americanum*)^21^. Furthermore, wild *Cebus capucinus* in Costa Rica, wild *Cebus olivaceus* in Venezuela, and semi-free-ranging *Sapajus* sp. in Brazil, anoint more during the wet season when there are more flying insects^19^,^23^,^24^.

Despite evidence for the medical efficacy of such substances used for anointing, non-medicinal explanations for the behaviour have also been proposed. Neotropical primates have well-developed olfactory communication, and many species scent mark substrates (Heymann 2006^27^ for a review). That scent marking can explain anointing in black-handed spider monkeys is evidenced by the observation that rubbing actions are limited to the chest, are more often performed by males, and are non-seasonal in their temporal distribution, despite seasonal differences in insect parasite abundance^5^,^6^. Similar hypotheses have been offered for anointing in capuchins, insofar as it may create a ‘group scent’^24^.

An interesting observation relevant to these functional hypotheses is that *Cebus*^19^,^24^,^28^,^29^,^30^,^31^, and owl monkeys *Aotus*^10^, often anoint ‘socially’, when one monkey rubs against another during anointing. However, there is mixed evidence for social anointing in *Sapajus*. Leca *et al.* (2007)^32^ and Paukner and Suomi (2008)^33^, found that their captive *Sapajus* rarely rubbed socially, and reported differences in the anointing behaviours of *Sapajus* and *Cebus*, hypothesizing that *Cebus* derives social benefits, such as the strengthening of social bonds, from fur rubbing, but that *Sapajus* does not. However, anting in *Sapajus* often involves numerous individuals^23^ leading Alfaro *et al.* (2012)^14^ to propose that the distribution and local abundance of anointing material determines the degree of sociality in capuchin anointing behaviours.

In a group of captive *Sapajus* sp. the frequency of aggression increased, and durations of affiliative behaviours decreased, following anointing with onion (*Allium cepa*)^33^. These findings are consistent with competition for resources. However, in a separate study, aggression towards animals that anointed in isolation also increased on their return to their group^34^, leading the authors to suggest a ‘chemo-signalling’ hypothesis in which there is a disruptive effect on olfactory communication. Since the effect was short-lived, we suggest it is also possible that dominant individuals might have been directing aggression at individuals that smelled of these resources as part of their normal despotic behaviour in controlling access to resources.

Regardless of the underlying functional reasons for anointing, there is undoubtedly a strong social element that has to be accounted for. Weeper capuchins (*Cebus olivacious*) anointed with millipedes ‘without competitive friction’ and age-sex classes that normally avoid each other came together to do so^19^. Individual *Cebus* might rub against other anointing monkeys simply to acquire the substance being applied, when resources are rare or at a low density in the wild (e.g. millipedes)^19^. Mutualism may also explain social anointing if it is a better way of covering hard-to-reach areas, such as between the shoulder blades, than individual anointing alone^31^, as has been shown for allogrooming in a range of primate species^35^.

Experiments on self-medicative behaviour in captive primates, outside of the context of disease, have been successfully employed to test hypotheses in greater detail than is possible in the wild^32^,^33^,^36^,^37^. Here we manipulate the abundance of an anointing resource given to two captive groups of *Sapajus* spp. to test key predictions derived from the main hypotheses for the functions of anointing and in particular, social anointing (Table 1).

Ticks and lice are partially controlled in primates by auto and social grooming^35^,^38^,^39^,^40^. If anointing treats such skin parasites in capuchin monkeys, as in the ‘medicinal hypothesis’ (Table 1), we might expect individual (self) anointing to target areas that an individual has difficulty grooming, such as those not visible to itself^35^,^40^, and furthermore social anointing should target areas that are difficult for an individual to reach physically. On the other hand, the ‘scent-marking hypothesis’ predicts that different age sex classes will anoint at different rates, and the behaviour will be restricted to different body parts, as in *Ateles*^5^.

If the function of social anointing is to strengthen social bonds as in the ‘social bonding’ hypothesis, we predict no difference in the proportion of social anointing to individual anointing when resources are abundant or rare. This prediction is shared by the ‘coordination of treatment’ hypothesis^41^ in which simultaneous medicinal treatment reduces re-infection of individuals, and by the ‘mutual application’ hypothesis^31^ in which social anointing treats hard-to-reach areas, such as between the shoulder blades. Conversely, the rare resource hypothesis^19^ predicts that because animals are socially anointing in order to obtain access to rare resources, social anointing will be rare when resources for anointing are abundant. Additionally, the ‘social bonding hypothesis’ predicts increased affiliative behaviour (e.g. grooming) following anointing sessions with more social anointing. The ‘rare resource’ hypothesis predicts that two monkeys that are *both* holding anointing resources will not form anointing dyads, whilst the ‘mutual application’ hypothesis predicts that monkeys holding anointing resources will continue to seek out other monkeys that hold resources, and that social anointing actions will target parts of the body that are inaccessible to a monkey rubbing individually.

We also address predictions generated by Paukner & Suomi’s^33^,^34^ observed changes in levels of aggression during and after anointing. The ‘chemo-signalling’ hypothesis, predicts changes in aggression during and after rubbing because odours in the resource mask natural chemo-signalling, so levels of aggression will be different when resources, and therefore odour, are rare or abundant. However, aggression could conceivably increase or decrease. On the other hand, the ‘dominance’ hypothesis predicts higher levels of aggression after bouts when there is more social rubbing and the ‘competition’ hypothesis predicts that there will be more aggression during bouts when there are fewer anointing resources, and that lower-ranking individuals will anoint less than higher-ranking individuals to avoid aggression.

## Study animals

The study was done at the University of St Andrews’ ‘Living Links to Human Evolution’ Research Centre in Edinburgh Zoo, Scotland (hereafter ‘Living Links’), with two similar groups of capuchin monkeys (*Sapajus* spp.), the ‘west’ group (n = 16; 1 dominant adult male, 3 subordinate adult males, 1 Subadult male, 3 Adult females, 1 subadult female, 4 Juvenile males, 1 Juvenile female, 2 infants) and ‘east’ group (n = 12; 1 dominant adult male, 3 subordinate adult males, 1 Subadult male, 3 Adult females, 1 Juvenile male, 1 Juvenile female, 2 infants). Age-sex classes were assigned before the study following Izawa (1980)^42^, which allows the identification of dominant adult males based on clearly defined physical and behavioural characteristics. The two groups are housed in near-identical, spacious enclosures with inside (190 m^2^) and outside (900 m^2^) sections. Both groups live in mixed species communities with common squirrel monkeys (*Saimiri* spp.) that are known to commonly form mixed species associations in the wild. For further information on Living Links and the monkeys studied see Leonardi *et al.* (2010)^43^, Bowler *et al.* (2011)^44^ and Macdonald & Whiten (2011)^45^.

## Method

Between July and November 2011, we replaced onions and related foods (garlic & leeks) from the diet with alternative foods. We tested each capuchin group on dry days twice a week by introducing pieces of onion to a defined area (approx 5 × 5 m) within the outside enclosure. We presented two experimental conditions on different occasions; a ‘rare-resource’ condition with one half of a large onion for each group, and an ‘abundant resource’ condition with half a large onion for each non-infant monkey in the group. We tested the groups one after the other in a randomly counterbalanced order. Successive sessions were separated by at least 48 hours. In each session, we completed one ‘focal individual follow’^46^, filming one focal individual with a video camera for 45 minutes from the introduction of the onion. We made one complete 45-minute focal follow for each non-infant monkey for each condition regardless of their anointing activity (a total of 28 sessions for the west group and 20 for the east group). Animals could move freely between the inside and outside enclosures and were filmed from the closest possible viewing points^45^ from distances of approximately 2–30 m.

Approval for the study was gained from the Ethics Committee of the University of St Andrews’ School of Psychology. Procedures were conducted in accordance with the guidelines of the Association for the Study of Animal Behaviour.

## Video coding

### Anointing behaviours

We defined ‘social anointing’ as anointing whilst in contact with another monkey, and ‘individual anointing’ as anointing without touching another individual. Using the video recording and the program *The Observer XT version 10, Noldus*, MB coded behaviour as in or out of view, in contact with other monkeys or not, in possession of a piece of onion or not, and anointing or not. Since anointing could involve frequent pauses, we defined an anointing bout as starting from the first rubbing action with onion, and finishing one minute after the last rubbing action.

**Figure 1:**
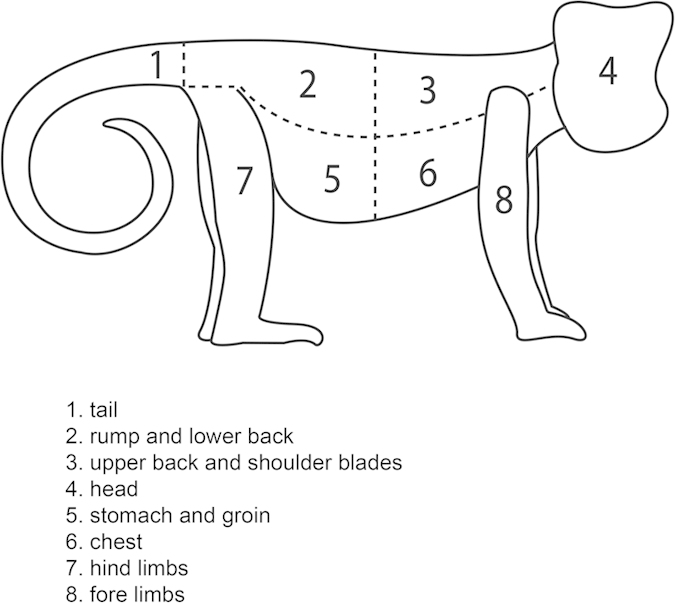
Capuchin monkey body parts coded to record anointing actions. *Illustration by Mark Bowler*.

We defined ‘rubbing actions’ as events where a body part or onion came into contact with and moved across the surface of another body part. We also recorded when carried babies were rubbed in this way. We discriminated eight regions of the capuchin body (Fig. 1) of similar surface area (following Zamma 2002^40^); the *tail, stomach and groin, chest, hind limbs* and *fore limbs* were defined as ‘accessible’ and ‘visible’ to the monkeys, the *rump and lower back*, and *head* were defined as ‘accessible’ and ‘non-visible’ and the *upper back and shoulder blades* was defined as ‘inaccessible’ and ‘non-visible’, because they could not reach the area with their hands or see the area themselves. One research assistant recorded the number of rubs on each body part from the videos. Because rubbing actions were sometimes very fast and sustained, variable visibility reduced the coders’ ability to accurately record the frequency of individual actions, and the monkeys frequently changed their position and body parts being rubbed, ‘1’ was scored for a body part at the start of any continuous sequence of rubbing actions on that body part. Each rubbing action was classified as ‘social’ when the focal monkey’s body part was rubbed by or on another monkey or onion held by it, or ‘individual’ when the body part was rubbed by the focal monkey’s own body part or onion. Thus ‘social rubs’ are actions within the behaviour of ‘social anointing’ as defined above.

### Aggression and affiliation

MB recorded all instances of aggression directed to or from the focal individuals for 45-minutes following the introduction of the onion, including:

#### Threaten

Open mouth, bared teeth, eyebrows raised and ears flattened, and direct staring towards another monkey, usually with rapid forward movements^47^, may include branch shaking or breaking and banging objects (pushed or pulled with hands, feet and/or tail). No physical contact is made.

#### Chase

Runs towards another monkey, displacing them with threats (see above) and/or aggressive vocalisations^47^, and without facial expressions associated with play^47^. No physical contact is made.

#### Attack

Contact, including biting, hitting, grabbing and pushing, accompanied by threats (see above) and/or aggressive vocalisations^47^, and without facial expressions associated with play^47^.

MB recorded the total time spent in social grooming^47^ immediately following anointing bouts for each focal monkey as a measure of affiliation. This allowed us to look for short-term, but not long-term changes in affiliation.

## Results

**Figure 2:**
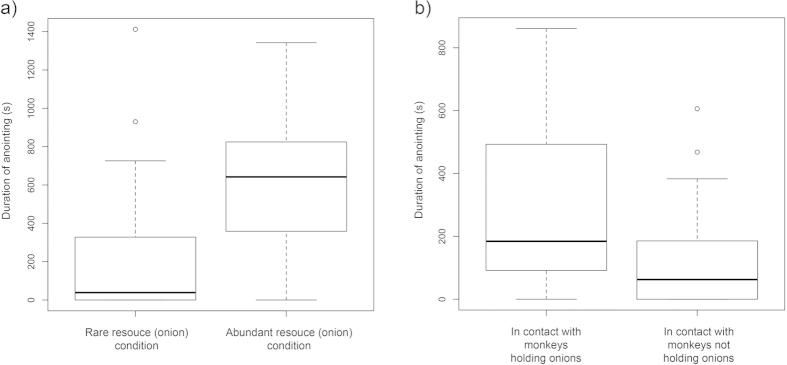
(**a**) Mean durations of anointing (including both individual and social anointing) in rare resource and abundant resource conditions in *Sapajus* sp. at Living Links. (**b**) Mean durations that focal monkeys holding onions spent in contact with other monkeys who also had or did not have onions, in the abundant resource condition.

**Figure 3:**
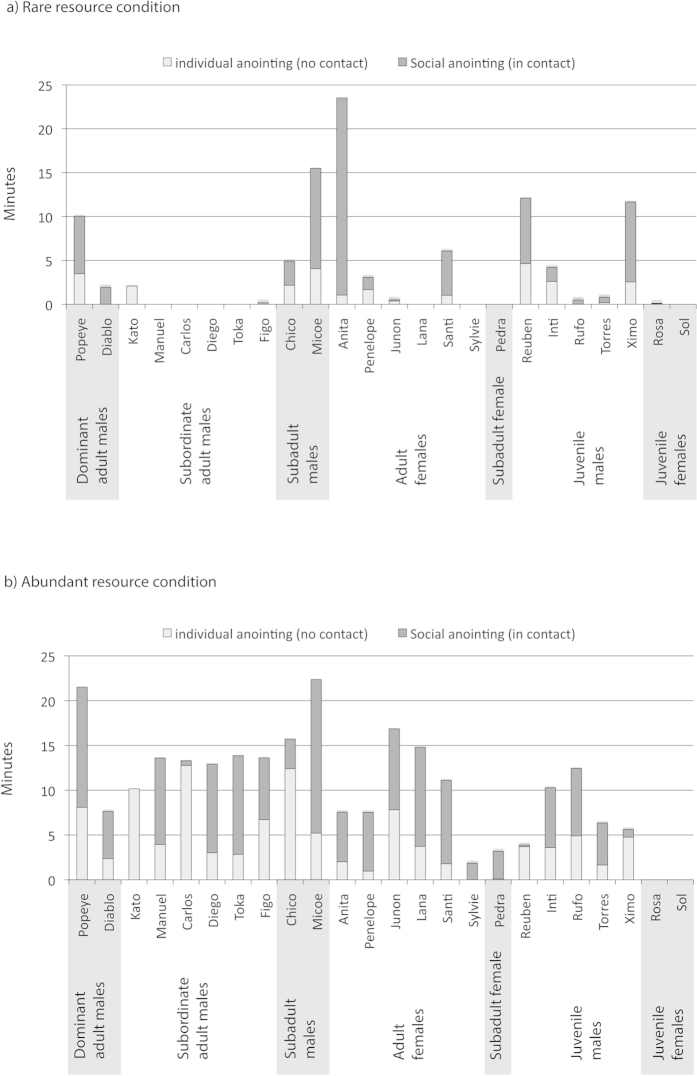
The relative durations of individual and social anointing in (a) rare resource and (b) abundant resource conditions for individuals in different age-sex classes.

Monkeys anointed (including individual and social anointing) for longer in the *abundant resource* condition (mean +/− SD = 615 s +/− 366 s, N = 24) than in the rare resource condition (mean +/− SD = 243 s +/− 371 s, N = 24) (ANOVA, F = 12.26, p = 0.00104) (Fig. 2a). Of the 24 focal individuals, 15 anointed in both conditions, one in only the *rare resource* condition, seven in only the *abundant resource* condition and one, a juvenile female, did not rub in either condition (Fig. 3). Only 2 out of 6 subordinate (non-alpha) adult males anointed in the *rare resource* condition, and those only for short durations. However, in the *abundant resource* condition, all subordinate adult males anointed at similar rates to other age-sex classes (mean +/− SD = 774.7 s +/− 83.0 s, N = 6, versus mean +/− SD = 666.5 s +/− 378.7 s, N = 8) (non-paired t-test, t = 0.6810, p = 0.509), indicating that the lower rates of anointing from this age-sex class in the rare resource condition were likely due to restricted access to resources.

For individuals that rubbed in both conditions, differences between the percentages of social anointing in *rare resource* (mean +/− SD = 66% +/− 29, N = 15) or *abundant resource* (mean +/− SD = 53% +/− 29, N = 15) conditions were not significant (matched pairs t-test, t = − 1.609, p = 0.130). Nor was there a significant difference between the percentages of social rubbing actions (see Methods) in *rare resource* (mean +/− SD = 26% +/− 29, N = 15) or *abundant resource* (mean +/− SD = 19% +/− 13, N = 15) conditions in individuals that anointed in both conditions (matched pairs t-test, t = − 1.098, p = 0.291). In the *rare resource* condition, fewer monkeys anointed, but those that did often scavenged pieces of onion from other anointing monkeys, and anointed both socially and individually.

In the *abundant resource* condition, where monkeys had the opportunity to choose whether to anoint socially or individually (on no occasion did a monkey take two onion halves at the same time, so resources were available for all individuals), focal individuals holding onions socially anointed more often in groups of other monkeys that held onions (mean +/− SD = 285.5 s +/− 265.1 s, N = 24) than with those that did not (mean +/− SD = 122.1 s +/− 166.9 s, N = 24) (ANOVA, F = 6.22, p = 0.0163) (Fig. 2b) indicating that the attraction was motivated by more than simply access to resources.

**Figure 4:**
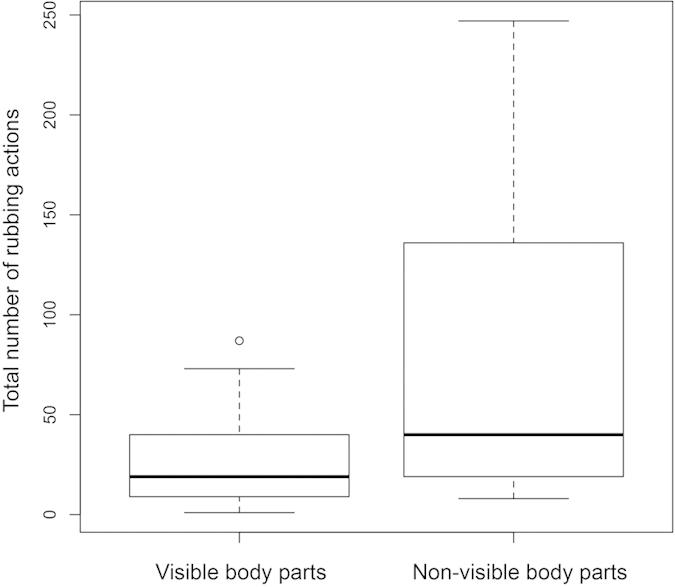
Total number of rubbing actions on ‘visible’ versus ‘non-visible’ body parts for individual rubbing actions during the abundant resource condition.

**Figure 5:**
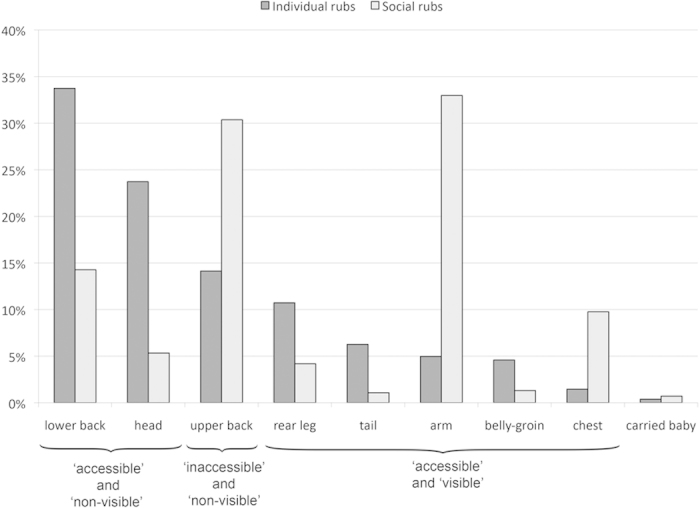
Percentages of ‘individual’ and ‘social’ rubbing actions on each body part and carried babies.

**Figure 6:**
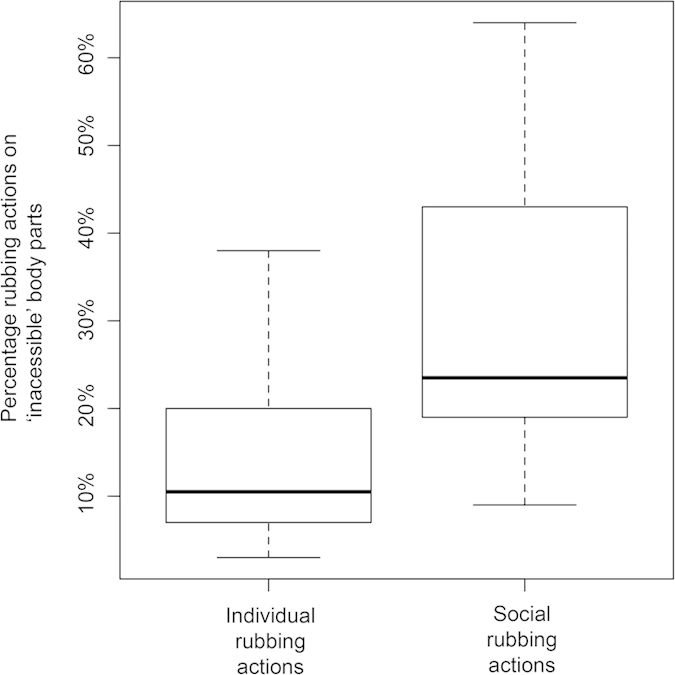
Percentages of rubbing actions on inaccessible body parts for individual and social rubbing actions, during the abundant resource condition.

Areas of the body that are non-visible to an individual monkey were subject to more rubbing actions (mean +/− SD = 77.8 +/− 73.4, N = 22) than body parts that are visible (mean +/− SD = 26.6 +/− 23.9, N = 22) (matched pairs t-test, t = − 4.456, p = 0.00011) (Figs 4 & 5). Individual and social anointing rubbing actions focused on different body parts (Fig. 5). Individual anointing focused on the head and lower back, whilst social anointing actions focused on the arms, upper and lower back and the chest. A greater percentage of social anointing rubbing actions were made on ‘inaccessible’ body parts (mean +/− SD = 30% +/− 17, N = 14) than for individual anointing rubbing actions (mean +/− SD = 14% +/− 10, N = 14) (matched pairs t-test, t = − 4.247, p = 0.00095) (Fig. 6). Notably, social rubbing actions reached dorsally between the shoulder blades (coded *within* the upper back category), an area that capuchin monkeys cannot reach with their hands (feet were only rarely used in anointing and by only a few individuals), thus only social rubbing achieved complete coverage of the body.

Aggression was rare overall (mean +/− SD = 0.05 events/minute +/− 0.06, N = 48), and there was no significant difference in the rates of aggression between the *rare resource* condition (mean +/− SD = 0.06 events/minute +/− 0.07, N = 24) and the *abundant resource* condition (mean +/− SD = 0.05 events/minute +/− 0.04, N = 24) (matched-pairs t-test: t = 0.651, df = 23, p = 0.52).

There was also no significant difference in time spent grooming between the *rare resource* condition (mean +/− SD = 181.5 s +/− 370.8 s, N = 24) and the *abundant resource* condition (mean +/− SD = 184.7 s +/− 385.1 s, N = 24) (ANOVA, F = 0.001, p = 0.977).

## Discussion

Anointing is an energetically costly behaviour that can last over 20 minutes in captive and wild *Cebus* and *Sapajus*^14^, considerably longer than any other recorded scent marking behaviour in any species of neotropical primate^27^. Wild capuchins may engage in anointing every two days^14^. Since the behaviour must be costly, we would expect it to provide significant benefits to the subjects. Olfactory communication has been suggested as one explanation for anointing^14^,^33^,^34^, but a convincing argument for what the animals are communicating remains to be offered. The mechanism by which anointing with strong smelling substances could positively affect olfactory communication, and thus improve an individual’s fitness, is not clear, unless rules as simple as ‘stronger smelling is more attractive’ dictate capuchin monkey olfactory communication systems. It has also been argued that capuchins might seek to mask their olfactory identity^33^, but in our study, all age-sex classes engaged in anointing, including dominant males, and occasionally young infants that were not subject to focal follows; both of these age-sex classes seem likely to benefit from a clear olfactory message of identity. Since olfactory signals evolved to benefit the signaller, it is hard to see how masking these could be advantageous in natural conditions. We saw no difference in time spent fur rubbing between age-sex classes, and monkeys’ anointed all body parts. Medicinal explanations for anointing are better supported by our results (Table 1); monkeys anointed areas that are non-visible to them more than areas that are visible to them. These areas are harder for an individual monkey to groom, so if anointing is an alternative therapy for ticks and lice, we might expect these areas to be targeted more, as we see in our experiments.

The social anointing behaviours in capuchin monkeys may have lead to the formulation of the social bonding hypotheses for the function of anointing in the species. The behaviour is certainly in need of explanation, since the benefits of anointing socially must outweigh not only the costs of sharing resources, but also the costs of potentially being subject to aggression in the case of subordinate monkeys, or of curbing aggression for dominant individuals. The *Sapajus* groups in our study anointed socially in every experimental session, and proportions of social rubbing relative to individual rubbing were high (53% in the abundant resource condition and 66% in the rare resource condition) relative to reports for both wild and captive *Cebus;* Curú (*C. capucinus*, wild) 54.7%^24^, Quepos (*C. capucinus*, wild) 42.9%^14^, Santa Rosa (*C. capucinus*, wild) 57.1%^14^, Bush Bush (*C. albifrons*, wild) 0%^14^, Masaguaral (*C. olivaceus*, wild) 46%^19^, Strasbourg (*C. capucinus*, captive) 49.3% with citrus, 15% with onions^19^. Bouts in our groups typically included salivating^48^ and tail coiling^19^, behaviours previously only observed in anointing *Cebus*, whilst overall rates of aggression were low. These results, along with observations of wild tufted capuchins anting^14^, and frequent informal observations of social anointing with lime fruits in our study group, before and after our experiments (Supplementary Electronic Resource 1), are at odds with many of the reported differences in anointing between these genera. Larger quantities of onion (and therefore odour) did not lead to increased aggression, and lower-ranking subordinate adult male monkeys anointed as much as dominant male and adult female monkeys when they had access to resources, so the ‘interference with olfactory communication’ hypothesis^33^,^34^ is not supported (Table 1).

Limiting the anointing resources did not lead to increased rates of aggression through competition. Competition for anointing materials, unlike competition for food, appears to be modulated by the benefits of sharing the resources. We found that anointing generally occurred with little aggression in our study groups. Our study groups were stable and had spacious enclosures, much like the captive *Cebus capucinus* in anointing studies^32^, which may have led to more natural anointing behaviour than in groups living in small enclosures^33^. We found no difference between the frequencies of social grooming immediately following bouts with more or less social anointing, which does not give us evidence for any short-term change in social behaviour. However longer-term changes could accumulate in groups that anoint frequently, and our tests do not exclude this possibility. Medium and long-term changes in affiliation after anointing could appropriately be measured using a social networking approach^49^.

The fact that there was no difference in the proportions of social anointing and individual anointing when resources were either abundant or rare, and the fact that individuals with materials continued to be attracted to other anointing individuals, suggest that there is more to social fur rubbing than individuals gaining access to rare resources^19^. Instead, the results showing that social anointing resulted in a more complete coverage of the body with anointing materials lends support to the mutual application hypothesis, in which social anointing leads to better coverage by medicinal substances (Table 1). Increased coverage on the upper back (and the upper arm or shoulder) may result from self-directed rubbing against other anointing individuals that may be a secondary source of material, but more frequent rubbing on the chest in social anointing results from individuals actively rubbing other individuals. The chest is very accessible to them, and is typically quickly saturated during anointing, since materials are held against the chest. Furthermore, young carried infants, that were not yet making anointing actions, were frequently anointed in this way. Thus, we report animals actively medicating other group members (see Supplementary Video S1), as observed for *Cebus capucinus* in Lomas Barbudal^31^.

Neither an anointer’s awareness of the medicinal action of materials, nor an intention to medicate, are implied by a functional medicinal explanation for anointing and social anointing. They may well be explained in terms of innate behaviours^50^, but alternatively, Meunier *et al.* (2008)^41^ showed that anointing in *Cebus capucinus* is a ‘collective behaviour with a mimetic underlying mechanism’. These authors suggest that synchronised anointing within a group could be advantageous to reduce re-infection rates, as with many parasite treatments. Social anointing might additionally facilitate the transfer of preferences for particular anointing materials, through social learning^14^, leading to learned differences between groups in wild populations^31^. We additionally propose that social anointing *physically treats* other individuals with the substances (*a la* Perry 2008^31^), with protective benefits to the self, through group hygiene and reduced re infection, and to likely kin in the group. We conclude that social anointing in capuchin monkeys is a form of mutual medication that improves coverage of topically applied anti-parasite medicines for both individuals and groups of capuchins.

## Additional Information

**How to cite this article**: Bowler, M. *et al.* Mutual medication in capuchin monkeys – Social anointing improves coverage of topically applied anti-parasite medicines. *Sci. Rep.* **5**, 15030; doi: 10.1038/srep15030 (2015).

## Supplementary Material

Supplementary Information

Supplementary Information

## Figures and Tables

**Table 1:** Hypotheses, predictions and results for the functions of anointing and social anointing in *Cebus* and *Sapajus*, and for the social behaviour of *Sapajus* during anointing.

Hypothesis		Description	Predictions	Results
Function of anointing	Scent marking hypothesis (Discussed in *Cebus* in Baker 1996 and in *Ateles*, Campbell, 2000).	Animals anoint with strong scents to communicate to group members or other groups	Different age-sex classes will anoint at different rates	NOT SUPPORTED During abundant resource conditions, all non-infant age-sex classes anointed at similar rates
			The behaviour may be restricted to specific body parts	NOT SUPPORTED All body parts were anointed
	Medicinal hypothesis (Baker 1996, Valderrama *et al.* 2000, Huffman 2007, Alfaro *et al.* 2011)	Primates apply strong smelling materials to their fur to obtain a medical benefit such as reduced skin parasite load or reduced parasitism from biting insects	There will be no difference in anointing rates between age-sex classes	SUPPORTED During abundant resource conditions, all non-infant age-sex classes anointed at similar rates
			All body parts will be covered	SUPPORTED All body parts were anointed
			Monkeys will anoint more on body parts that are not visible to them and are therefore harder to groom	SUPPORTED Monkeys anointed more on body parts that are not visible to them
Function of social anointing	Social bonding hypothesis (Baker, 1996, Leca *et al.*, 2007, Paukner & Suomi 2008)	Animals strengthen social bonds by engaging in anointing behaviours in contact with group members	There will be no difference in the proportion of social anointing to individual anointing when resources are abundant or rare	SUPPORTED There was no difference in the proportions of social anointing (in time or number of actions) between the rare and abundant resource conditions
			Monkeys will groom more immediately following sessions with more social anointing	NOT SUPPORTED There was no significant difference between grooming rates immediately after rare resource (less social anointing) and abundant resource (more social anointing) conditions.
	Rare resource hypothesis (Valderrama *et al.* 2000)	Individuals without items are obtaining chemicals from the bodies of others because they do not have direct access to resources	Social anointing will be much rarer, as a proportion of all anointing, when the anointing resource is abundant	NOT SUPPORTED There was no difference in the proportions of social anointing (in time or number of actions) between the rare and abundant resource conditions
			Groups of monkeys in which more than one individual has an anointing resource should be rare	NOT SUPPORTED Individuals holding onions socially anointed more often in groups of other monkeys that held onions
	Coordination of treatment hypothesis (Meunier *et al.* 2008)	Optimizes medicinal treatment by reducing group parasite load and therefore re infection of individuals	There will be no difference in the proportion of social anointing to individual anointing when resources are abundant or rare	SUPPORTED There was no difference in the proportions of social anointing (in time or number of actions) between the rare and abundant resource conditions
	Mutual application hypothesis (Perry 2008)	Treats hard-to-reach areas, such as between the shoulder blades, obtaining better coverage of topically applied medicines	There will be no difference in the proportion of social anointing to individual anointing when resources are abundant or rare	SUPPORTED There was no difference in the proportions of social anointing (in time or number of actions) between the rare and abundant resource conditions
			Animals holding anointing material will continue to seek out other anointing animals, and groups of monkeys in which more than one individual has an anointing resource will be common	SUPPORTED Individuals holding onions socially anointed more often in groups of other monkeys that held onions
			Social anointing will target parts of the body that are inaccessible to an individual monkey and therefore achieve more complete coverage	SUPPORTED Social rubbing actions on ‘inaccessible’ body parts were more frequent than on ‘accessible’ body parts.
Social behaviour of *Sapajus* during anointing	Chemo-signalling hypothesis (Paukner & Suomi 2008)	Aggression increases during and after rubbing because odours in the resource mask natural chemo-signalling in the capuchins	Levels of aggression will be different when resources, and therefore odour, are rare or abundant	NOT SUPPORTED Levels of aggression did not differ significantly when resources were rare or abundant.
	Competition hypothesis (Perry 2008, Paukner & Suomi 2008)	Aggression increases during and after rubbing through competition for access to resource pieces	Lower-ranking individuals should anoint less than higher-ranking individuals to avoid aggression from higher-ranking individuals	PARTIALLY SUPPORTED Subordinate adult males anointed infrequently in the rare resource condition, but frequently in the abundant resource condition
			Fewer pieces of resource should create more competition, and therefore more aggression, than more pieces	NOT SUPPORTED There was no significant difference in the rates of aggression between the rare resource condition and the abundant resource condition
	Dominance hypothesis	Increased aggression results from individuals re-affirming dominance relationships before and after the unusually close-proximity behaviour	There will be more aggression when there is more social rubbing	NOT SUPPORTED There was no significant difference in the rates of aggression between the rare resource (less social anointing) and abundant resource (more social anointing) conditions

It should be noted that the hypotheses are largely non-exclusive.

## References

[b1] Reiger I. Scent rubbing in carnivores. Carnivora 2, 17–25 (1979).

[b2] D'Havé H., Scheirs J., Verhagen R. & De Coen W. Gender, age and seasonal dependent self-anointing in the European hedgehog *Erinaceus europaeus*. Acta theriol, 50, 167–173 (2005).

[b3] Hart B. L., Clayton D. H. & Moore J. Behavioural defence. in Host-parasite evolution: general principles and avian models. (eds. Clayton D. & Moore J. ) 59–77 (Oxford, 1997).

[b4] Birkinshaw C. R. Use of millipedes by black lemurs to anoint their bodies. Folia Primatol 70, 170–171 (1999). 10394067 10.1159/000021691

[b5] Campbell C. J. Fur rubbing behavior in free-ranging black-handed spider monkeys (*Ateles geoffroyi*) in Panama. Am. J. Primatol. 51, 205–208 (2000). 10902669 10.1002/1098-2345(200007)51:3<205::AID-AJP5>3.0.CO;2-L

[b6] Laska M., Bauer V. & Salazar L. T. H. Self-anointing behavior in free-ranging spider monkeys (*Ateles geoffroyi*) in Mexico. Primates 48, 160–163 (2007). 17103123 10.1007/s10329-006-0019-9

[b7] Morrogh-Bernard H. C. Fur-rubbing as a form of self-medication in *Pongo pygmaeus*. Int. J. Primatol. 29, 1059–1064 (2008).

[b8] Guidorizzi C. E. & Raboy B. E. Fur-rubbing with plant exudates in wild golden-headed lion tamarins (*Leontopithecus chrysomelas*). Am. J. Primatol. 71, 75–75 (2009).

[b9] Zito M., Evans S. & Weldon P. J. Owl monkeys (*Aotus* spp.) selfanoint with plants and millipedes. Folia Primatol 74, 159–161 (2003). 12826735 10.1159/000070649

[b10] Jefferson J. P., Tapanes E. & Evans S. Owl monkeys (*Aotus* spp.) perform self- and social anointing in captivity. Folia Primatol. 85, 119–134 (2014). 24852183 10.1159/000359970

[b11] Silva J. Especiacao nos macacos-prego e caiararas, genero *Cebus* Erxleben, 1777 (Primates, Cebidae). PhD Thesis, Universidade Federal do Rio de Janeiro, Rio de Janeiro, Brazil (2001).

[b12] Alfaro J. W. L., Silva J. D. S. E. & Rylands A. B. How different are robust and gracile capuchin monkeys? An argument for the use of *Sapajus* and *Cebus*. Am. J. Primatol. 74, 273–286 (2012). 22328205 10.1002/ajp.22007

[b13] Hershkovitz P. Mammals of northern Colombia. Preliminary report No. 4: Monkeys (Primates), with taxonomic revisions of some forms. Proceedings of the United States National Museum 98, 323–427 (1949).

[b14] Alfaro J. W. L. *et al.* Anointing variation across wild capuchin populations: a review of material preferences, bout frequency and anointing sociality in *Cebus* and *Sapajus*. Am. J. Primatol. 74, 299–314 (2012). 21769906 10.1002/ajp.20971

[b15] Eisenberg J. F. & Kleiman D. G. Olfactory communication in mammals. Annu. Rev. Ecol. Syst. 3, 1–32 (1972).

[b16] Brockie R. Self-anointing by wild hedgehogs, *Erinaceus Europaeus*, in New Zealand. Anim. Behav. 24, 68–71 (1976).

[b17] Weldon P. J., Aldrich J. R., Klun J. A., Oliver J. E. & Debboun M. Benzoquinones from millipedes deter mosquitoes and elicit self-anointing in capuchin monkeys (*Cebus* spp.). Naturwissenschaften 90, 301–304 (2003). 12883771 10.1007/s00114-003-0427-2

[b18] Huffman M. A. & Vitazkova S. K Primates, plants, and parasites: the evolution of animal self-medication and ethnomedicine. in Ethnopharmacology (eds. Elisabetsky E. & Etkin N. L. ), 367–389 (Eolss, 2007).16987625

[b19] Valderrama X., Robinson J. G., Attygalle A. B. & Eisner T. Seasonal anointment with millipedes in a wild primate: a chemical defence against insects. J. Chem. Ecol. 26, 2781–2790 (2000).

[b20] Peschke K. & Eisner T. Defensive secretion of the tenebrionid beetle, *Blaps mucronata*: Physical and chemical determinants of effectiveness. J. Comp. Phys. 161, 377–388 (1987). 10.1007/BF00603963 3668879

[b21] Weldon P. J. *et al.* Anointing chemicals and hematophagous arthropods: responses by ticks and mosquitoes to Citrus (*Rutaceae*) peel exudates and monoterpene components. J. Chem. Ecol. 37, 348–359 (2011). 21409496 10.1007/s10886-011-9922-7

[b22] Carroll J. F., Kramer M., Weldon P. J. & Robbins R. G. Anointing chemicals and ectoparasites: effects of benzoquinones from millipedes on the lone star tick, *Amblyomma americanum*. J. Chem. Ecol. 31, 63–75 (2005). 15839480 10.1007/s10886-005-0974-4

[b23] Verderane M. P. *et al.* Anting in a semifree-ranging group of *Cebus apella*. Int. J. Primatol. 28, 47–53 (2007).

[b24] Baker M. Fur rubbing: use of medicinal plants by capuchin monkeys (*Cebus capucinus*). Am. J. Primatol. 38, 263–270 (1996).

[b25] Aboelhadid S. M., Kamel A. A., Arafa W. M. & Shokier K. A. Effect of *Allium sativum* and *Allium cepa* oils on different stages of *Boophilus annulatus*. Parasitol. Res. 112, 1883–1890 (2013). 23435922 10.1007/s00436-013-3344-0

[b26] Corzo-Martínez M., Corzo N. & Villamiel M. Biological properties of onions and garlic. Trends Food Sci. Tech. 18, 609–625 (2007).

[b27] Heymann E. W. Scent marking strategies of New World primates. Am. J. Primatol. 68, 650–661 (2006). 16715511 10.1002/ajp.20258

[b28] Baker M. Identification and selection of fur rubbing materials by white-faced capuchin monkeys (*Cebus capucinus*). Am. J. Primatol. 42, 93 (1997).

[b29] Buckley J. S. The feeding behavior, social behavior, and ecology of the white-faced monkey *Cebus capucinus* at Trujillo, Northern Honduras, Central America. PhD Thesis, University of Texas at Austin (1983).

[b30] Panger M. A. Object-use in free-ranging white-faced capuchins (*Cebus capucinus*) in Costa Rica. Am. J. Phys. Anthropol. 106, 311–321 (1998). 9696147 10.1002/(SICI)1096-8644(199807)106:3<311::AID-AJPA4>3.0.CO;2-P

[b31] Perry S. Manipulative monkeys: the capuchins of Lomas Barbudal (Harvard University Press, 2008).

[b32] Leca J. B., Gunst N. & Petit O. Social aspects of fur-rubbing in *Cebus capucinus* and *C. apella*. Int. J. Primatol. 28, 801–817 (2007).

[b33] Paukner A. & Suomi S. J. The effects of fur rubbing on the social behavior of tufted capuchin monkeys. Am. J. Primatol. 70, 1007–1012 (2008). 18626908 10.1002/ajp.20595 PMC2562658

[b34] Paukner A. & Suomi S. J. Social after-effects of fur rubbing in tufted capuchin monkeys (*Cebus apella*): increased antagonism and reduced affiliation. Primates 53, 297–301 (2012). 22350275 10.1007/s10329-012-0300-z PMC3638249

[b35] Barton R. Grooming site preferences in primates and their functional implications. Int. J. Primatol. 6, 519–532 (1985).

[b36] Huffman M. A. & Hirata S. An experimental study of leaf swallowing in captive chimpanzees - insights into the origin of a self-medicative behavior and the role of social learning. Primates 45, 113–118 (2004). 15095043 10.1007/s10329-003-0065-5

[b37] Huffman M. A., Spiezio C., Sgaravatti A. & Leca J. Leaf swallowing behavior in chimpanzees (*Pan troglodytes*): biased learning and the emergence of group level cultural differences. Anim. Cogn. 13, 871–880 (2010). 20602132 10.1007/s10071-010-0335-8

[b38] Hutchins M. & Barash D. P. Grooming in primates: implications for its utilitarian function. Primates 17, 145–150 (1976).

[b39] Boccia M. L. A functional analysis of social grooming patterns through direct comparison with self-grooming in rhesus monkeys. Int. J. Primatol. 4, 399–418 (1983).

[b40] Zamma K. Grooming site preferences determined by lice infection among Japanese macaques in Arashiyama. Primates 43, 41–49 (2002). 12091746 10.1007/BF02629575

[b41] Meunier H., Petit O. & Deneubourg J. L. Social facilitation of fur rubbing behavior in white-faced capuchins. Am. J. Primatol. 70, 161–168 (2008). 17823917 10.1002/ajp.20468

[b42] Izawa K. Social behavior of the wild black-capped capuchin (*Cebus apella*). Primates 21, 443–467 (1980).

[b43] Leonardi R., Buchanan-Smith H. M., Dufour V., MacDonald C. & Whiten A. Living together: behavior and welfare in single and mixed species groups of capuchin (*Cebus apella*) and squirrel monkeys (*Saimiri sciureus*). Am. J. Primatol. 72, 33–47 (2010). 19790191 10.1002/ajp.20748

[b44] Bowler M. T., Buchanan-Smith H. M. & Whiten A. Assessing public engagement with science in a university primate research centre in a national zoo. PloS one 7, e34505 (2012). 22496822 10.1371/journal.pone.0034505 PMC3319593

[b45] Macdonald C. & Whiten A. The ‘Living Links to Human Evolution’ Research Centre in Edinburgh Zoo: a new endeavour in collaboration. International Zoo Yearbook 45, 7–17 (2011).

[b46] Altmann J. Observational study of behavior: sampling methods. Behaviour, 49, 227–266 (1974). 4597405 10.1163/156853974x00534

[b47] Freese C. H. & Oppenheimer J. R. The capuchin monkeys, genus *Cebus*. inEcology and Behavior of Neotropical Primates Vol. 1 (eds. Coimbra-Filho A. F. & Mittermeier R. A. ) 331–390 (Academia Brasileira de Ciencias, 1981).

[b48] Fragaszy D. M., Visalberghi E. & Fedigan L. M. The complete capuchin (Cambridge University Press, 2004).

[b49] Whitehead H. Analyzing animal societies : quantitative methods for vertebrate social analysis (Chicago, University of Chicago Press, 2008).

[b50] de Roode J. C., Lefèvre T. & Hunter M. D. Self-medication in animals. Science 340, 150–151 (2013). 23580516 10.1126/science.1235824

