# Supplementary material for: Mutual medication in capuchin monkeys – Social anointing improves coverage of topically applied anti-parasite medicines
Source: Sci Rep. 2015 Oct 12;5:15030. doi: 10.1038/srep15030 (PMC4601033; doi:10.1038/srep15030)
Supplement: Supplementary Information [file srep15030-s1.pdf]

Supplementary Video S1 for 'Mutual medication in capuchin monkeys – Social anointing improves coverage of topically applied anti-parasite medicines'

Mark Bowler, Emily J. E. Messer, Nicolas Claidière & Andrew Whiten

Supplementary Video S1 Legend

Tufted capuchin monkeys (*Sapajus* sp.) in the Living Links to Human Evolution Research Centre, Edinburgh Zoo, Scotland. Clips: Subadult male anointing individually; Social anointing with spring onions; Adult male and subadult male anointing socially with limes; Adult male anointing onto carried infant; Large group of eight anointing capuchins.
